# A Complementary and Revised View on the N-Acylation of Chitosan with Hexanoyl Chloride

**DOI:** 10.3390/md19070385

**Published:** 2021-07-02

**Authors:** Berthold Reis, Niklas Gerlach, Christine Steinbach, Karina Haro Carrasco, Marina Oelmann, Simona Schwarz, Martin Müller, Dana Schwarz

**Affiliations:** 1 Leibniz-Institut fuer Polymerforschung Dresden e.V., Hohe Straße 6, 01069 Dresden, Germany; reis@ipfdd.de (B.R.); gerlach@ipfdd.de (N.G.); steinbach@ipfdd.de (C.S.); haro@ipfdd.de (K.H.C.); oelmann@ipfdd.de (M.O.); simsch@ipfdd.de (S.S.); mamuller@ipfdd.de (M.M.); 2 Department Chemistry and Food Chemistry, Technische Universität Dresden, 01062 Dresden, Germany

**Keywords:** hexanoyl chitosan, chitosan modification, substitution degree, pH variation

## Abstract

The modification of the biobased polymer chitosan is a broad and widely studied field. Herein, an insight into the hydrophobization of low-molecular-weight chitosan by substitution of amino functionalities with hexanoyl chloride is reported. Thereby, the influence of the pH of the reaction media was investigated. Further, methods for the determination of the degree of substitution based on ^1^H-NMR, FTIR, and potentiometric titration were compared and discussed regarding their accuracy and precision. ^1^H-NMR was the most accurate method, while FTIR and the potentiometric titration, though precise and reproducible, underlie the influence of complete protonation and solubility issues. Additionally, the impact of the pH variation during the synthesis on the properties of the samples was investigated by Cd^2+^ sorption experiments. The adjusted pH values during the synthesis and, therefore, the obtained degrees of substitution possessed a strong impact on the adsorption properties of the final material.

## 1. Introduction

Chitosan and modifications thereof have been the target of a vast number of studies [1,2,3,4,5,6]. The driving force behind the great number of publications is the biodegradability of chitosan to non-toxic products, the high abundance of the raw biomaterial chitin, and the primary amino group yielding in various types of applications such as the medical sector, environmental protection, wastewater treatment, and agriculture, only to name some of the largest [7,8,9]. Thus, the deacetylation of chitin to chitosan is now performed on a large industrial scale. The determination of the degree of deacetylation, which defines the properties, has already been studied in detail by Weißpflog et al. [10]. However, one major drawback for the application of chitosan as an absorber is its solubility at low pH.

To further improve and overcome those challenges, the properties of the fascinating polymer chitosan, various functional groups have been substituted to the primary amino group on the polymer backbone [11]. However, often the synthesis and subsequent analysis of such substitution reactions are rather complicated due to the amount of different functional groups in the polymer. In the current study, we focus on the N-acylation of chitosan attaching a hexanoyl group (also called caproyl chloride) to the amino functionality. The hexanoyl group on the chitosan backbone reduces the solubility of the polymer in acidic media, making it a suitable adsorber material at low pH ranges. Various synthetic routes have been reported, including the use of hexanoic acid anhydrides [7,12], hexanoic acid combined with carbodiimide hydrochloride [13], or hexanoyl chloride [14,15]. The majority is based on the use of organic solvents and higher temperatures. We chose the substitution with hexanoyl chloride conducted in water at room temperature, which minimizes costs. For the nucleophilic substitution reaction with hexanoyl chloride, the pH of the aqueous solution needs to be thoroughly adjusted. Thus far, for the substitution of hexanoyl chloride on chitosan, the pH of the reaction solution has always been adjusted to a pH of 7 [14,16,17].

In this study, we show that the substitution degree is extremely sensitive to the pH of the solution and therefore needs to be carefully adjusted in order to obtain reproducible products. The issue of profound characterization of the modified chitosan has also been addressed in several studies [10,11,18]. Thereby, several innovative methodologies using picric acid [19] or ninhydrin essay [20] have been developed. Herein, we focus on standard analytics, in particular ^1^H-NMR, IR-spectroscopy, and potentiometric titration. The substitution degree was calculated with all three methods, and the comparability, as well as reliability, were discussed. Additionally, we investigated the influence of the initial pH on the spatial structure. Further, we conducted proof-of-concept adsorption experiments with CdSO_4(aq)_, which displayed the significant influence even small pH changes during the reaction synthesis have on the final adsorption performance.

## 2. Results and Discussion

**Scheme 1:**
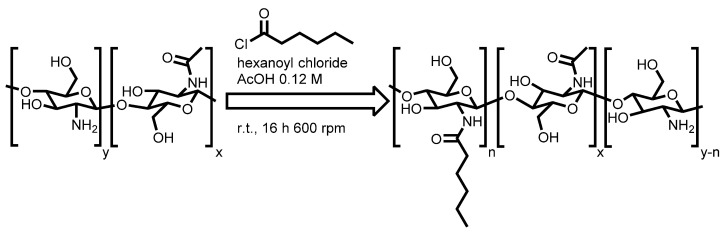
Reaction scheme of the hexanoyl substitution on chitosan.

Low molecular weight chitosan with an acetylation degree (DS%_ACETYL_) of 10% was modified with hexanoyl chloride. The synthesis is described in detail in Section 3.2. In brief, Ch90/60/A1 was dissolved in 0.12 M acetic acid, and the pH of the reaction solution was carefully adjusted to pH 6.0, 6.5, 7.0, 7.5, and 8.0, respectively. Hexanoyl chloride was added dropwise to the solution and was stirred for 16 h at r.t. Subsequently, the mixture was neutralized, poured into acetone (see images in Supporting Information (SI), Figure S1), filtered, and washed with methanol at 60 °C. In the course of the reaction, the amino functionality was partially substituted by the acyl chloride (Scheme 1). All yielded products are referred to as Hexanoyl-chitosan (H-chitosan), and for specification, the pH value is added (e.g., H-chitosan-7.5). The appearance of the samples varies with the increasing pH of the reaction solution, from compact agglomerates to finer particles (see Figure S2). This indicates variations in chemical (e.g., DS%) and physical properties (e.g., TGA, Viscosity, XRD).

### 2.1. Characterization of the H-Chitosan Samples

To determine the chemical composition and the degree of substitution (DS%, constitutional repeating unit n in Scheme 1) of the modified chitosan ^1^H-NMR, FTIR-spectroscopy measurements, zeta potential, and potentiometric titration were conducted. The DS% was calculated for each method. The obtained values were compared and discussed. Further, the influence of the substitution on the physical properties was investigated by XRD, viscosity experiments, and nitrogen sorption as well as DLS for colloidal stability measurements.

**Figure 1:**
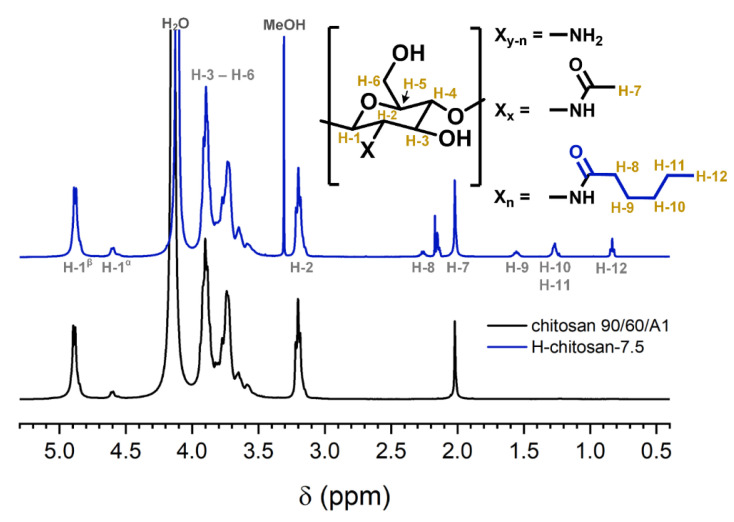
^1^H NMR spectra of native chitosan 90/60/A1 (black) and H-chitosan-7.5 (blue) with peak assignments to corresponding protons (H-1^α^ and H-1^β^ for anomeric structures, see Scheme S3). The ^1^H NMR spectra of H-chitosan-6.0, H-chitosan-6.5, H-chitosan-7.0, H-chitosan-7.5, and H-chitosan-8.0 are shown in Figure S3.

For the analysis with ^1^H-NMR, Ch90/60/A1 and the five H-chitosan samples were dissolved in 0.1 M DCl in D_2_O solution (Figure 1). All shifts were assigned to their corresponding protons in accordance with the literature [21]. The peaks at 4.89 ppm and 4.52–4.67 ppm are attributed to the H-1^β^ and the H-1^α^ proton of the deacetylated and acylated glucosamine in all spectra, respectively. Further, both native and the H-chitosan samples exhibited similar peaks in the region of 3.50–4.00 ppm assigned to the protons ranging from H-3 to H-6 and the H-2 bond to amid functionalities. The H-2 coupled to an amino functionality is shifted upfield to 3.12–3.28 ppm. The CH_3_ peak related to the native acetylation of the chitosan is located at 2.02 ppm. Through integration and referencing to the H-1 peak areas, an acetylation degree (DS%_Acetyl_) of 10% was calculated for Ch90/60/A1 (Equation (1), Section 3.4.1), confirming the data of the provider (DS%_ACETYL_ = 10%). The spectra of the H-chitosan samples further show hexanoyl-related peaks at 0.83 ppm (–CH_3_), 1.27 ppm (different –CH_2,_) and 1.54 ppm (–CH_2_). The peak at 2.26 ppm originates from the –CH_2_ group nearest to the carbonyl and is therefore subjected to proton–deuterium exchange (peaks between 2.12 and 2.18 ppm).

For the five H-chitosan samples, the DS%_NMR_ was calculated by using the peak areas of H-1^β^ and the H-1^α^, since these signals are not subjected to overlap (Table 1, Equation (2), Section 3.4.1). The NMR spectra with integrals are given in the supporting information (Figure S3).

The increase in the DS%_NMR_ when increasing the pH of the reaction media from 6.0 to 7.5 can be explained by the increased neutralization of the byproduct hydrochloric acid at higher pH values, which shifts the reaction equilibrium toward the desired product. This effect seems to cease when surpassing the pH value of 7.5 due to the larger part of precipitated chitosan, which takes more time to dissolve while the pH drops during the course of the reaction. Furthermore, this kinetically favors the hydrolysis of hexanoyl chloride to hexanoic acid as a side reaction (see Scheme S1). Thus, a higher pH value should accelerate this hydrolysis. For a precise NMR analysis and subsequent DS%_NMR_ calculation, the existence of potential byproducts such as hexanoic acid needs to be precluded. However, in the ^1^H NMR spectra, no carboxylic acid peak corresponding to the potential byproduct hexanoic acid at 2.16 ppm, 1.28 ppm, and 0.86 ppm was observed [22]. These hexanoic acid peak shifts slightly differ from their corresponding CH_2_ or CH_3_ group in the hexanoyl functionality. The spin multiplicity of the hexanoyl functionality was well resolved, suggesting that no corresponding peaks of hexanoic acid are underlying those of the attached functionalization. Therefore, no residual hexanoic acid from the hydrolysis (see Scheme S1) remained within the samples.

Further, the potential cleavage of the amide under the conditions within the NMR tube (90 °C, 0.1 M DCl) (see Scheme S2) can be precluded. Additionally, it is known that such reactions only occur in severe conditions, e.g., with acids in significantly higher concentrations [23]. Hence, NMR is a very valid and accurate method to determine the DS% of H-chitosan samples.

A complementary method for the determination of functional groups and the DS is attenuated total reflection (ATR) Fourier transform infrared (FTIR) spectroscopy. Thereby, two methods and calculation strategies were applied and compared.

ATR-FTIR spectra on the powdered samples were recorded and evaluated according to the method (i.e., method 1) of Le Tien et al. [14], which is based on a study of Moore et al. [24]. For both H-chitosan samples and the native chitosan, prominent bands between 3600 and 3100 cm^−1^ ν(OH) due to hydroxyl groups and bound water, at 2918 cm^−1^ and 2879 cm^−1^ ν(CH_2_) due to hydrocarbon moieties, at 1655 cm^−1^ (shoulder) and 1588 cm^−1^ (Amide I and II, respectively), at 1510 cm^−1^ (δ(NH_3_^+^)) due to ammonium groups and at 1080 cm^−1^ (ν(C–O)) due to C–O–H and C–O–C linkages in polysaccharides were obtained (Figure S4).

The DS%_FTTR-1_ was calculated according to Le Tien et al. [14] (see method 1, Equation (3), Section 3.4.2), yielding a DS% _FTTR-1_ value of 19.9% for H-chitosan-7.5. Thus, the determined DS% _FTTR-1_ value is more than six times larger than the DS%_NMR_ value. The other H-chitosan samples feature a similar trend. It has to be noted that this evaluation method is based on the ratio of the Amide I band and the ν(OH) band. The Amide I overlaps with δ(OH) of water (1640 cm^−1^), and the ν(OH) is composed of contributions of hydroxyl groups of chitosan and bound water. Both influences are inclined to falsify the calculation.

**Figure 2:**
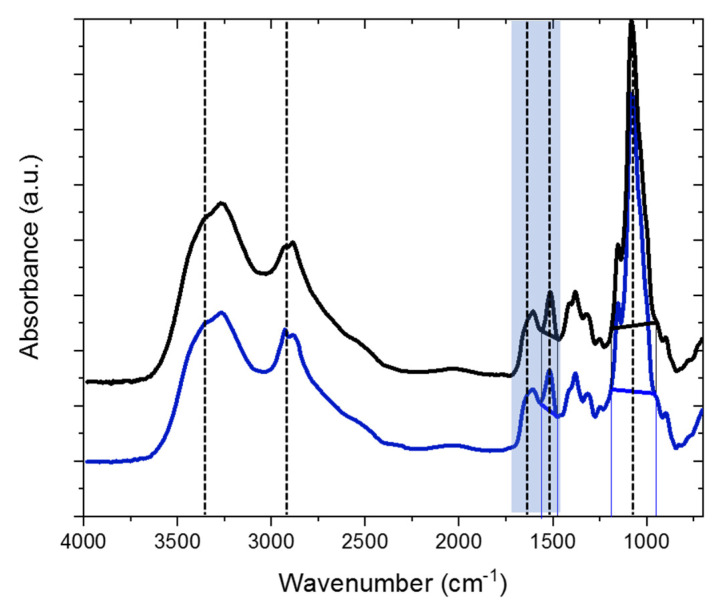
ATR-FTIR spectra of thin films of native chitosan 90/60/A1 (black) and H-chitosan-7.5 (blue) with diagnostic IR bands (left to right): νOH, νCH_2_, Amide I, δ(NH_3_^+^), ν(C-O), blue area subjected to LSA (all H-chitosan spectra in Figure S5).

Based on the results from method 1, ATR-FTIR spectra of all samples were recorded again by casting a thin film of each sample on the ATR crystal (Figure 2 for H-chitosan-7.5, Figure S5 for all). To reduce the influence of the water band, the film was carefully dried and subsequently constantly purged with nitrogen during measurement. Since thin films are more accessible for drying through nitrogen than compact powders are, with this way of sample preparation, the exclusion of water can be ensured more effectively. For the evaluation of the thin film ATR-FTIR spectra, a modified method (method 2) was followed, which is based on the ratio (Q) between integrals of the Amide I band at 1655 cm^−1^ (determined via line shape analysis (LSA) Figure S6) and the ν (C–O) band at 1080 cm^−1^ (Q = I_Amide I_/I_SACC_). The ratio of the native chitosan (Q_CHIT_) was determined and a normalization factor (*n*) was calculated by using the known acetylation degree (DS%_ACETYL_) of 10%. The corresponding (higher) ratio Q_H-CHIT_ of the H-chitosan samples is used to calculate the total substitution degree (DS%_TOTAL_), which includes the hexanoyl and the acetyl-related Amide I signals. The substitution degree DS%_FTIR-2_ was determined from the difference DS%_TOTAL_ − DS%_ACETYL_ (Equation (7), Section 3.4.2) and is displayed in Table 1. In general, the yielded DS%_FTIR-2_ values correlated with the DS%_NMR_ with an average deviation of ±1.0% (Table 1, Figure S7). The majority of the DS%_FTIR-2_ were above the values determined by NMR. Presumably, there is still residual water in the dried film samples, and the corresponding δ(OH) band intensity might contribute to that of the Amide I band. Nonetheless, this novel method poses a significant improvement to method 1 with several advantages. First, it is not based on the questionable ν(OH) intensities (see above). Second, it uses line-shape analysis for the determination of the integral of the Amide I band. Further, the thin films are more susceptible to nitrogen drying. Altogether, this leads to an improved reduction of falsifying water influences. Therefore, this procedure can be considered as a suitable method for fast overview measurements.

Thermogravimetric analysis showed a bimodal disintegration for the low molecular weight (M_w_ = 90–150 kDa) native chitosan, with the first decomposition step ranging from about 180 °C to 390 °C and the second ranging from 390 °C to 470 °C (Figure S8). The H-chitosan samples exhibited a similar primary decomposition step ranging from 180 °C to 580 °C. However, the second step differs from that of the native chitosan, ranging up to 650 °C. Further, within this second step, a third and smaller one is located within the H-chitosan functions. This step ranges from 420 °C to 475 °C and is larger for the higher DS%. Overall, the thermal stability was increased by the substitution.

**Figure 3:**
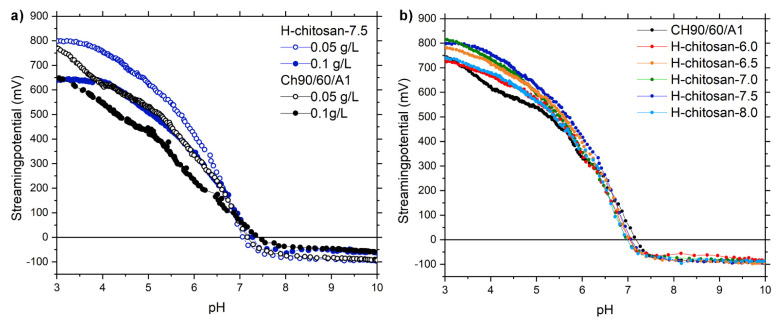
Streamingpotential vs. pH for (**a**): native chitosan 90/60/A1 at 0.05 g/L (black, open sphere), 0.1 g/L (black, full sphere), H-chitosan-7.5 at 0.05 g/L (blue, open sphere), and at 0.1 g/L (blue, full sphere). Streamingpotential vs. pH at 0.05 g/L for (**b**): native chitosan 90/60/A1 (black), H-chitosan-6.0 (red), H-chitosan-6.5 (orange), H-chitosan-7.0 (green), H-chitosan-7.5 (blue), H-chitosan-8.0 (light blue).

Further, the zeta potential in the dependency of the pH was investigated. This method provides information on the surface charge at specific pH values, which is of great importance for absorber materials, for example. To test the influence of the ionic strength, a similar approach for titration was chosen. By dissolute the initial 1 g/L solution with a pH of 1, solutions of 0.05 g/L and 0.1 g/L were obtained for native chitosan and H-chitosan-7.5, respectively. The streamingpotential vs. pH curves are shown in Figure 3. In Figure 3a, the isoelectric point (IEP) for native chitosan and H-chitosan-7.5 for both concentrations (i.e., 0.05 g/L and 0.1 g/L) was located around pH 7.2. Additionally, the different H-chitosan samples were compared using only 0.05 g/L solutions (Figure 3b). All IEPs are located at 7.0 ± 0.1. This indicates that low absolute deviations in DS% of 1–2% have little to no influence on the IEP.

Another fast and versatile method to determine the DS%_Pot_ is potentiometric titration, whereby positively charged amino functionalities are complexed by the short polyanion sodium polyethylene sulfonate (NaPES). Lin et al. provided an equation (Equation (8), Section 3.4.3) to calculate the DS%_Pot_ from the volume of the consumed NaPES [25]. To investigate the influence of pH and ionic strength, different dissolutions from the initial solution of 1 g/L at pH_0_ 1 were prepared. Thus, three solutions with concentrations of 0.01 g/L, 0.05 g/L, and 0.1 g/L were prepared, and their respective pH_dis_ were recorded. The calculated DS%_Pot_ are displayed in Table 2. The dissolution of the initial H-chitosan-7.5 solution yielded different pH_dis_ values for the three different concentrations. The calculated DS%_Pot_ for the three solutions varies by ±0.1%, which shows a low influence of the pHids. This is due to the already fully protonated chitosan, as shown in the streamingpotential vs. pH curve. Both charge densities of the chitosan 90/60/A1 and the H-chitosan-7.5 show consistency within concentrations of 0.05 and 0.10 g/L. At 0.01 g/L, the concentration was not high enough for accurate measurements. Thus, 0.05 g/L was chosen for the following more extensive investigations.

For all H-chitosan samples, the observed DS%_Pot-1_ was significantly higher (5.6–8.1% higher) than the corresponding DS%_NMR_ (compare Table SI 1). This may be based on the hindrance of the complex arrangement through “trapping” of the amino functionalities within hydrophobic domains. The increase in hydrophobicity leads to a reduction of the solubility by reducing the number of protonable amino functionalities and enabling self-aggregation in the solution. Although this may be beneficial regarding applications such as drug delivery, it complicates potentiometric titration as an analytical method. Commonly, native chitosan dissolved with HCl_(aq)_ at r.t. with a magnetic stirrer for 24 h. The optimum pH to dissolve H-chitosan is at pH 1 (after stirring 24 h at r.t.). However, since this modified chitosan is poorly soluble, there are some larger particles in the solution, which are visible to the naked eye. For potentiometric titration, the solubility of the samples is a significant factor. Hence, to investigate this parameter by improving the solubility, in a second attempt, the samples were stirred at 50 °C for 24 h and then treated with an ultrasonic bath for 2 h. The DS%_Pot-2_ was calculated via the same equation (Equation (8), Section 3.4.3) and compared in Table 3.

The DS%_Pot-2_ values were, on average, about 0.9% closer to the DS%_NMR_ determined with NMR. Nevertheless, the determined values are still about two to three times as high as the DS%_NMR_, which is why this method again is more suitable for quick overview measurements than profound investigation.

**Figure 4:**
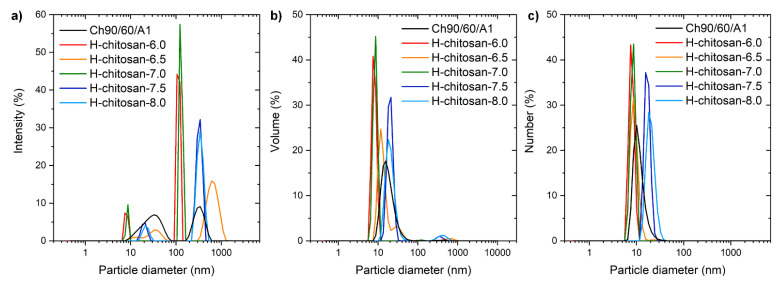
DLS-measurements of chitosan and H-chitosan samples (1 g/L, at pH = 1, solved at 50 °C for 24 h with additional ultrasonic treatment for 2 h) of native chitosan 90/60/A1 (black), H-chitosan-6.0 (red), H-chitosan-6.5 (orange), H-chitosan-7.0 (green), H-chitosan-7.5 (blue), H-chitosan-8.0 (light blue) with (**a**): intensity distribution (**b**): volume distribution and (**c**): number distribution.

To further investigate the self-aggregation properties in solution, all solved samples were investigated by dynamic light scattering (DLS) measurements. For the samples solved at r.t. for 24 h, the distributions are depicted in Figures S9–S11, whereby the larger, optically visual, non-solved particles were not captured by the method. The samples solved using the improved method (50 °C, 24 h, ultrasonic bath for 2 h) are shown in Figure 4. All chitosan samples featured a bimodal size distribution in the intensity curve, as shown in Figure 4a. The smaller particles were in the range of 10–40 nm in diameter, and the larger particles possessed particles diameters of several hundred nanometers. However, the volume and number curves clearly show just the small particles yielding in an extremely small ratio of larger particles shown in the intensity curve. Thus, the average particle sizes according to the number distribution were around 8 nm for H-chitosan-6.0, H-chitosan-6.5, and H-chitosan-7.0; around 10 nm for Ch90/60/A1; and around 18 nm for H-chitosan-7.5 and H-chitosan-8.0. H-chitosan-6.0, H-chitosan-6.5, and H-chitosan-7.0 featured smaller particles in comparison to the native chitosan because the solubility of the H-chitosan samples decreased. H-chitosan-7.5 and H-chitosan-8.0 exhibited the highest DS% values, resulting in an increased agglomeration number of polymer chains per particle to further compensate for the decreased solubility. It is already known that H-chitosan samples form self-aggregated particle structures in aqueous conditions [26]. The influence of the pH of the solution on the particle size has already been studied by Pa and Yu [27]. The DLS intensity curves at pH 1.55 are in agreement with the size distribution shown in Figure 4.

To investigate the spatial structure in a dry state, nitrogen sorption experiments and X-ray diffraction measurements were conducted (compare Section 3.4.5 and Section 3.4.8). Thereby no significant specific surface area (SSA) was found either for the native chitosan or for the H-chitosan samples (Figure S12). Further, all X-ray diffraction patterns of the H-chitosan samples showed the same broad reflex at 2Θ = 20° as native chitosan (compare Figure S13), which indicated a long-range order and coincided with the literature [14]. A deviation is the small shoulders between 25° and 30° 2Θ, which indicates a minor proportion of differently structured domains within the H-chitosan samples. Overall, both nitrogen sorption and X-ray diffraction suggest that the dry state structure of native and modified chitosan are very similar.

Viscosity and rheology properties were determined using an oscillating rotational rheometer (Section 3.4.9). As seen in Figure S14, the H-chitosans differ from native chitosan at lower velocity gradients of 0.01 1/s to 5.30 1/s. In this region, the H-chitosans flow, exhibiting more pronounced viscoelastic characteristics, whilst the native chitosan maintains more of a plateau at about 0.04 Pa. A possible explanation is the breaking-up of the hydrogen bonds between the chitosan backbones through the hydrophobic hexanoyl moiety. The low substitution degrees would explain why no stabilization via hydrophobic domains took place. This coincides with the observation that all H-chitosan exhibit very similar functions. Even the smallest DS% of 1.6% for H-chitosan-6.0 decreases the shear stress by several orders in magnitude.

### 2.2. Adsorption Experiments with CdSO_4(aq)_

**Figure 5:**
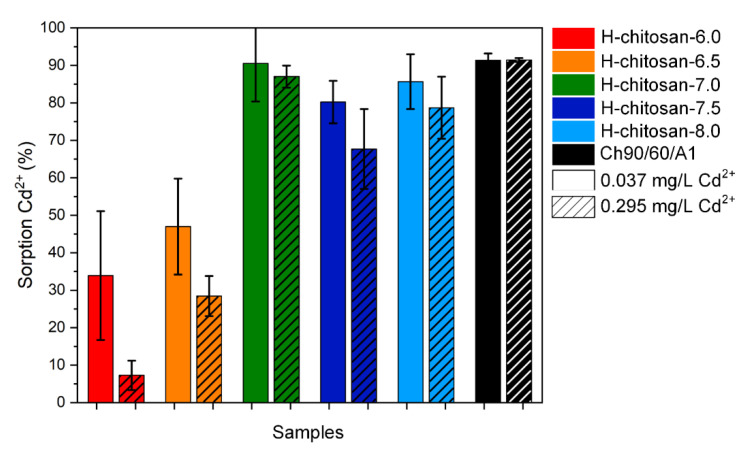
Adsorption of the different H-chitosan samples using the initial Cd^2+^ concentrations of 0.037 mg/L (plain) and 0.295 mg/L (striped). The corresponding pH values of the solution before and after the adsorption as well as the pH values of the H-chitosan samples in ultrapure water are shown in Figure S15.

Chitosan is an extremely good adsorber for water treatment applications. Thus, chitosan adsorbs, for example, heavy metal ions in very high amounts [6]. To show the influence of the DS% on the adsorption properties of H-chitosan, we investigated the adsorption of Cd^2+^ on the five diverse H-chitosan samples for two different concentrations. The H-chitosan samples were treated with initial concentrations of 0.037 mg/L and 0.295 mg/L Cd^2+^, which corresponds to the 10- and 100-fold value of the drinking water concentration limit [28]. H-chitosan-7.0, H-chitosan-7.5, and H-chitosan-8.0 featured the highest DS%_NMR_ values and thus also higher adsorption rates for Cd^2+^ with values up to 90% Cd^2+^ removal. Among those three H-chitosan samples, the DS%_NMR_ values vary marginally, yielding similarly high adsorption values. In comparison, H-chitosan-6.0 with the lowest DS%_NMR_ value exhibited the lowest Cd^2+^ removal rates (Figure 5). The native chitosan features very good adsorption properties for Cd^2+^ removal with a distinct smaller standard deviation due to the homogeneity of the sample. Cd^2+^ is removed by chitosan due to complexation with the primary amino groups on the chitosan backbone [29]. Thus, although the samples are solid in this investigation, chitosan is swelling in water to a relatively large extent, which leads to a better complexation process with Cd^2+^. For comparison, the H-chitosan exhibited limited swelling properties (see viscosity measurements, Figure S14) due to the hexanoyl group. Furthermore, as shown in the DLS measurements, the particle size and agglomeration number of polymer chains per particle are dependent on the DS%_NMR_, resulting in different sorption properties.

Thus, even subtle differences in synthesis conditions, such as a pH difference of 0.5, cause significant deviations in application; here, there is a 50–70% difference in adsorption of Cd^2+^ for H-chitosan-6.5 to H-chitosan-7.0.

## 3. Materials and Methods

### 3.1. Materials

The chitosan 90/60/A1 (origin crab shell, M_w_ = 90–150 kDa, manufacturer’s specification) was purchased from Biolog Heppe^®^ GmbH (Landsberg, Germany) and used without further purification. The indexes relate to a deacetylation degree of 90%, a viscosity of 60 mPas, and 1% ash content. Hexanoyl chloride, hydrochloric acid (37%), sodium hydroxide, and cadmium sulfate were purchased from Sigma Aldrich (Taufkirchen, Germany) and used without any further purification. Concentrated acetic acid was purchased from Merck KGaA (Darmstadt, Germany), whilst methanol and acetone were purchased from VWR (99%) (Darmstadt, Germany). As adsorption and reaction media ultrapure water obtained by a Milli-Q Advantage A10^®^ system from Merck KGaA (Darmstadt, Germany), (TOC 5 ppb, resistivity of 18.2 MΩ*cm at 25 °C) was used.

### 3.2. Synthesis of H-Chitosan

The synthesis is based on the method reported by Le Tien et al. [14]. A unit of 1.3 g of the purchased 90/60/A1 chitosan was dissolved for 16 h in 175 mL 0.12 M acetic acid. The pH value of the solution was adjusted to 6.0, 6.5, 7.0, 7.5, or 8.5 in separate experiments, with 2 M NaOH creating a slurry of partially precipitated chitosan. Special focus was directed towards this pH adjustment due to the longer equilibration times (1–2 h) of the system. Subsequently, hexanoyl chloride was added drop-wise with a mass ratio of 2:1 hexanoyl chloride/chitosan to the mixture, which was then stirred at 600 rpm for 16 h at r.t. The pH of the clear reaction mixture was again adjusted to about 7 and then poured into 2 L of acetone. The slurry was filtered and washed with boiling methanol. The resulting product was then dried in an oven at 50 °C for several hours.

### 3.3. Adsorption Experiments with CdSO_4_

100 mg of the modified chitosan was weighted in a 50 mL centrifuge tube. A total of 30 mL of Cd^2+^ solution (0.03 mg/L and 0.3 mg/L, respectively) was added. After stirring 24 h with a magnetic stirrer at 400 rpm, the samples were centrifuged for 8 min at 10,000 rpm. Each sample was tested with one concentration of Cd^2+^ at least six times. Based on the six measured concentrations per sample, the standard deviation was calculated and shown as error bars in Figure 5. A unit of 8 mL of the supernatant was offset with 2 mL of 20 wt% nitric acid for ICP-OES analysis. In addition, the pH of the supernatant was measured.

### 3.4. Characterization and Analysis

#### 3.4.1. H-NMR Measurements

The chitosan samples were dissolved 0.1 M DCl in D_2_O. Care was taken to ensure that the samples were dispersed completely. ^1^H-NMR was measured using an Avance III 500 MHz Bruker Biospin system.

The acetylation degree (DS%_ACETYL_) was calculated using the integral of the CH_3_ (I_CH3_) group at 2.02 ppm divided through the number of its protons (3) and the integral of the H-2 proton (I_H-2_) at 3.12–3.28 ppm (Equation (1)).

DS%_ACETYL_ = I_CH3_/(3 × I _H-1β + H-1α_) × 100 (1)

The substitution degree DS%_NMR_ was calculated using the sum of three integrals related to the hexanoyl residue (I_CH3 0.8 ppm_; I_2xCH2 1.3 ppm_; I_CH2 1.5 ppm_) dived by the number of participating protons (9) and the integral of the H-1^β^ and H-1^α^.

DS%_NMR_ = (I_CH3 0.8 ppm_ + I_2xCH2 1.3 ppm_ + I_CH2 1.5 ppm_)/(9 × I _H-1β + H-1α_) × 100 (2)

#### 3.4.2. FTIR Measurements

##### Method 1

Powder ATR-FTIR measurements (method 1) were conducted with a Tensor 27 (Bruker Corporation, Billerica, MA, USA) device equipped with a Platinum ATR module (Bruker Corporation). ATR-FTIR spectra from powdered samples were measured by pressing either chitosan or H-chitosan-7.5 against a single reflection diamond within the Golden Gate attachment (Specac, UK), which are given in Figure S4.

To determine the substitution degree DS%_FTIR-1_, the procedure of Le Tien [14] adapted from Moore et al. [24] was applied, which is based on the intensity ratio of Amide I band of N-acetyl groups and ν(OH) band of hydroxyl groups of chitosan. For the evaluation of DS%, the following equation was used (method 1):

DS%_FTTR-1_ = A_1655_/A_3450_ − 0.27 × 100 (3)

Thereby A_1653_ and A_3450_ are the absorbances at 1655 cm^−1^ and 3450 cm^−1^, respectively, and 0.27 specifies the acetylation in native chitosan.

##### Method 2

Thin films of either native chitosan or H-chitosan-, respectively, were casted onto Ge internal reflection elements (IRE, 50 × 20 × 2 mm^3^) from their respective solutions (50 µL) in 0.001 M HCl, dried at 50 °C, placed in a dedicated in situ cell and purged by N_2_ stream. The samples were measured in solid state with a resolution of 2 cm^−1^ and with 100 scans (method 2).

In method 2, modified from Moore et al. [24], the DS% was determined based on thin chitosan films applying a line-shape analysis procedure ranging from 1700 cm^−1^ to 1475 cm^−1^ to determine the integral of the Amide I band. Thereby, this spectral section was fitted using three band components (1656 cm^−1^, 1611 cm^−1^, 1511 cm^−1^) with 50% Gaussian and 50% Lorentz lineshape, respectively, and a baseline correction was undertaken (Figure S5). The DS%_FTIR-2_ can be calculated based on the integral ratio between the Amide I component (1656 cm^−1^) due to acetylated/hexanoylated amino groups and the integral of ν(C-O) band due to hydroxyl and ether linkages of polysaccharides (denoted saccharide band), respectively.

In the measured ATR-FTIR spectra, the ratios (Q) of the integrals of the Amide I band at 1656 cm^−1^ (I_Amide I,_ LSA) and the saccharide band from 1200–950 cm^−1^ (I_SACC_) were calculated for native chitosan (CHIT) and hexanoylated chitosan (H-CHIT), respectively (Equation (4)).

I_Amide I CHIT_/I_SACC CHIT_ = Q_CHIT_; I_Amide I H-CHIT_/I_SACC H-CHIT_ = Q_H-CHIT_ (4)

It was found by Moore et al. [24] that the Q_CHIT_ is proportional to the known acetylation degree (DS%_ACETYL_, here 10%) and can therefore be normalized to DS%_ACETYL_ via a factor *n* (Equation (5)).

DS%_ACETYL_ = Q_CHIT_ × *n* × 100 = 10% (5)

Taking the same value for factor n and assuming similar absorption coefficients for Amide I band related to both hexanoylated and acetylated amino groups the total degree of acetylation DS%_ACETYL,_ and hexanoylation DS%_HEX_ can be analogously calculated (Equation (6)).

DS%_TOTAL_ = DS%_ACETYL_ + DS%_HEX_ = Q_H-CHIT_ × *n* × 100 = *X*% ≥ 10% (6)

(Assuming no trans-amidification (replacing acetyl by hexanoyl groups)) DS%_FTIR-2_ can be calculated by subtracting DS%_ACETYL_ from DS%_TOTAL_ according to Equation (7).

DS%_FTIR-2_ = DS%_HEX_ = DS%_TOTAL_ − DS%_ACETYL_ (7)

#### 3.4.3. Potentiometric Titration and Zetapotential vs. pH

By stirring the chitosans in 2% acetic acid for 24 h, a 1 g/L solution was prepared. In additional cases, the solution was prepared by stirring at 50 °C for 24 h and subsequently treated in an ultrasonic bath for 2 h. The pH of around 3 was then adjusted to pH 1 with 1 M HCl. These solutions were subsequently diluted to 0.01 g/L, 0.05 g/L, and 0.1 g/L solutions with ultrapure water. A unit of 10 mL of each solution was titrated against NaPES (0.001 M) in a particle charge detector MÜTEK PCD-04 from the company BTG Instruments GmbH in Wessling, Germany. The DS%_Pot_ was determined using the equation of Lin et al. [25] with ΔV_Ch_ and ΔV_HCh_ being the consumed volumes of the polyanion for native chitosan and H-chitosan, respectively:

DS%_Pot_ = (ΔV_Ch_ − ΔV_HCh_)/(ΔV_Ch_ + 0.609 × ΔV_HCh_) × 100 (8)

For the streaming potential vs. pH, the solutions were titrated against a 0.1 g/L NaOH from their initial pH after dissolution (compare Table 2) up to a pH of 10. The zeta potential was measured again using the MÜTEK PCD-04.

#### 3.4.4. Inductively Coupled Plasma Optical Emission Spectrometry (ICP-OES)

ICP-OES (iCAP 7400 from Thermo Scientific, Waltham, USA) was used to determine the heavy metal ion (Cd^2+^) and sulfate ion concentrations in simulated water. The standards used were: standard 1: Cd (5 mg/L); S (5 mg/L); standard 2: Cd (1 mg/L); S (1 mg/L). A 4-point calibration was carried out. Therefore, the standards were diluted 1:2, 1:4, and 1:8.

#### 3.4.5. Nitrogen Sorption Studies

Nitrogen sorption studies were performed using the Autosorb iQ MP from Quantachrome Instruments. All samples were dried in a vacuum oven at 90 °C for 24 h. The samples were activated by degassing in vacuum (5 × 10^−10^ mbar) at 90 °C for another 20 h. The nitrogen sorption measurements were performed at 77 K with 100–150 mg sample. The acquired data were fitted using Brunauer Emmett Teller method (BET).

#### 3.4.6. Dynamic Light Scattering

Dynamic light scattering was measured using a Zetasizer ZS device from Malvern Panalytical GmbH, Kassel, Germany. The same solutions which were subjected to the potentiometric titration and zeta potential vs. pH measurements were investigated. The solutions were additionally heated at 50 °C for 18 h and dissolved 2 h in the ultrasonic bath. The refractive index of the polymer was set to 1.530.

#### 3.4.7. pH Measurement

pH measurement was carried out with the device SevenExcellence from Mettler Toledo (Gießen, Germany) at room temperature.

#### 3.4.8. X-ray Diffraction Measurements (XRD)

For XRD measurements, the chitosan powders were spread out on a Si low background sample holder (Bruker Corporation, Billerica, MA 01821, USA) and measured using a D8 DISCOVER (Bruker, Billerica, MA 01821, USA) equipped with a VÅNTEC 500 2D detector. Each sample was measured from two positions (1: Θ = 10° and Det. = 15°; 2: Θ = 10° and Det. = 40°) for 30 min, respectively, and the resulting diffractograms were merged.

#### 3.4.9. Oscillating Rotational Rheometer Measurements

The rotational rheometer measurements were conducted using a HaakeTM MarsTM Rheometer (Thermo Fisher Scientific Inc., Waltham, MA, USA). H-chitosan or native chitosan solutions of 1 g/L with pH = 1 were prepared and measured at 20 °C using a plate-plate PP60° Ti system with a gap of 0.3 mm. A range of 0.01 1/s to 8000 1/s was measured by increasing and then decreasing the γ in 40 steps, respectively.

#### 3.4.10. Thermogravimetric Analysis (TG)

TG was performed by using the device 1 Star System from Mettler Toledo, Gießen, Germany. The measurements were carried out with approximately 7–8 mg of chitosan in a platinum crucible. The investigated temperature range was from 25 °C to 1000 °C with a heating rate of 10 °C/min under air atmosphere with a flow rate of 40 mL/min.

## 4. Conclusions

Within this study, the degree of substitution (DS%) of the five H-chitosan samples, prepared at different pH values in the reaction mixture, was precisely calculated based on NMR spectroscopy. Based on the calculated DS%_NMR_, the pH values of the reaction mixture had a strong influence on the final DS% of the product and thus on the final properties. The determination of the DS% for the H-chitosan using the commonly reported ATR-FTIR spectroscopy-based method did not lead to a coinciding result with the NMR measurements. This can be explained by the water influence on the ν(OH) band (3450 cm^−1^) and the Amide I band (1655 cm^−1^). A new method using line shape analysis for the Amide I and referencing the polysaccharide band (1080 cm^−1^) proved to render a better result, with a general deviation of ±1.0% of the DS%_FTIR-2_ to DS%_NMR_ over all H-chitosans. Investigation with potentiometric titration showed that the DS%_Pot_ determined are significantly higher (average 6.7%) than the corresponding DS%_NMR_. This is presumably due to self-aggregation and solubility issues. An alteration of the standard solvation method leads to a reduction of this average value to 5.8% higher than DS%_NMR_. Through DLS measurements, the self-aggregation in solution was confirmed. Further, a decrease in viscosity was observed between solutions of native chitosan to H-chitosan. The properties in the dry state were investigated by nitrogen sorption and XRD, but little to no differences were made out between modified and unmodified material.

In several overview experiments, the influence of the synthesis of the H-chitosan samples on the adsorption performance of Cd^2+^ ions was demonstrated. Whilst H-chitosan-6.0 adsorbed the lowest amount of Cd^2+^, the removal by H-chitosan-7.0, H-chitosan-7.5, and H-chitosan-8.0 was between 75% and 90%. These findings illustrate how even subtle differences in synthesis cause significant deviations in application-oriented properties.

## Figures and Tables

**Table 1:** NMR- and FTIR-based calculation of the substitution degree DS% for the H-chitosan samples.

H-Chitosan	DS%_NMR_ *	DS%_FTIR-1_ **	DS%_FTIR-2_ ***
H-chitosan-6.0	1.6%	16.6%	2.9%
H-chitosan-6.5	2.1%	20.8%	1.8%
H-chitosan-7.0	2.7%	20.1%	5.3%
H-chitosan-7.5	3.0%	19.9%	3.8%
H-chitosan-8.0	2.9%	19.5%	3.0%

* DS%_NMR_ calculated with H-1 proton signals (H-1^α^ + H-1^β^) as reference; ** DS%_FTIR-1_ calculated using method 1 (Equation (3), Section 3.4.2); *** DS%_FTIR-2_ calculated using method 2 (Equations (4)–(7), Section 3.4.2).

**Table 2:** Titration of chitosan 90/60/A1 and H-chitosan-7.5 with NaPES for two different concentrations and respective pH values. The stock solution featuring an initial pH of 1 (pH_0_) was diluted with ultrapure water yielding in the pH_dis_.

Concentration	pH_0_	pH_dis_ 90/60/A1	pH_dis_ H-Chitosan-7.5	c (mmol/L) 90/60/A1	c (mmol/L) H-Chitosan-7.5	DS%_Pot-1_
0.01 g/L	1.0	2.9	2.9	6.2	5.4	8.7%
0.05 g/L	1.0	2.2	2.2	5.8	5.0	8.6%
0.10 g/L	1.0	2.0	2.0	5.7	5.0	8.8%

**Table 3:** Determined DS%_Pot_ of the H-chitosan solutions (0.05 g/L) with different methods of dissolving chitosan at pH 1 in HCl_(aq)_.

H-Chitosan	DS%_NMR_	DS%_Pot-1_ *	DS%_Pot-2_ **
H-chitosan-6.0	1.6%	8.2%	6.9%
H-chitosan-6.5	2.1%	9.8%	9.9%
H-chitosan-7.0	2.7%	10.8%	11.1%
H-chitosan-7.5	3.0%	8.6%	7.7%
H-chitosan-8.0	2.9%	8.5%	6.0%

* Samples solved over 24 h at r.t.; ** samples solved over 24 h at 50 °C with subsequent ultrasonic treatment (2 h).

## Data Availability

The data presented in this study are available in the Supporting Information and upon request from the corresponding author.

## Supplementary Materials

The following are available online at https://www.mdpi.com/article/10.3390/md19070385/s1, Figure S1. Images of the precipitation in acetone (a) and the gel-like product after filtering and washing with methanol (60 °C) (b) (here H-chitosan-6.0). Figure S2. Images of the dried products H-chitosan-6.0 (a), H-chitosan-6.5 (b), H-chitosan-7.5 (c), H-chitosan-8.0 (d). The lower pH values tend to form more compact fragments. Scheme S1. Hydrolysis of hexanoyl chloride as competing reaction to chitosan acylation. Scheme S2. Potential cleavage of hexanoyl functionality within the NMR tube (according to the NMR spectra, the reaction did not occur). Figure S3. ^1^H-NMR spectra of chitosan (black), H-chitosan-6.0 (red), H-chitosan-6.5 (orange), H-chitosan-7.0 (green), H-chitosan-7.5 (blue), H-chitosan-8.0 (light blue) and the respective integrals when H-2 = 1.000. Scheme S3. Different glycosidic linkages within chitosan backbone leading to the two ^1^H-NMR signals H-1^β^ and H-1^α^. Figure S4. ATR-FTIR spectra (powder, 100 scans, 2 cm^−1^ resolution) of native chitosan 90/60/A1 (black) and H-chitosan-7.5 (blue). Figure S5. Thin film ATR-FTIR spectra (method 2) of chitosan (black), H-chitosan-6.0 (red), H-chitosan-6.5 (orange), H-chitosan-7.0 (green), H-chitosan-7.5 (blue), H-chitosan-8.0 (light blue). Figure S6. Line shape analysis of H-chitosan-7.5 with measured spectrum (black), total fit (green, rms = 0.00088), Amide I (red), Amide II (yellow), δNH_3_^+^ (light blue), baseline (dark blue). Figure S7. Correlation of DS%_FTIR-2_ and DS%_NMR_ with a maximum absolute deviation of 2.6% and an average deviation of 1.0%. DS%_FTIR-2_ tends to render higher DS% than those determined via NMR. The black line indicates ideal correlation of DS%_FTIR-2_ and DS%_NMR_. Figure S8. Thermogravimetric analysis of chitosan (black), H-chitosan-6.0 (red), H-chitosan-6.5 (orange), H-chitosan-7.0 (green), H-chitosan-7.5 (blue), H-chitosan-8.0 (light blue). Table S1. Substitution degrees of H-chitosans determined via potentiometric titration and difference to DS%_NMR_. Figure S9. DLS intensity distribution measurements (1 g/L, pH 1) of native chitosan 90/60/A1 (black) and H-chitosan-7.5 (blue). The distribution of the H-chitosan-7.5 shows smaller assemblies (500 nm–1 μm) and larger aggregates (5 μm–10 μm) whilst chitosan only exhibits pristine polymer particles. Further, in all H-chitosan solutions, small, unsolved particles were visible that are too large in order to be measured with DLS. Figure S10. DLS volume distribution measurements (1 g/L, pH 1) of native chitosan 90/60/A1 (black) and H-chitosan-7.5 (blue). The distribution of the H-chitosan-7.5 shows smaller assemblies (500 nm–1 μm) and larger aggregates (5 μm–10 μm), while chitosan only exhibits pristine polymer particles. Further, in all H-chitosan solutions, small, unsolved particles were visible that are too large in order to be measured with DLS. Figure S11. DLS number distribution measurements (1 g/L, pH 1) of native chitosan 90/60/A1 (black) and H-chitosan-7.5 (blue). In all H-chitosan solutions, small, unsolved particles were visible, that are too large in order to be measured with DLS. Figure S12. Nitrogen sorption measurements. Neither native chitosan 90/60/A1 (black) nor H-chitosan (e.g., H-chitosan-7.5, blue) exhibited significant specific surface areas (S_BET_ < 3 m²/g).; Figure S13. X-ray diffraction measurements of chitosan (black), H-chitosan-6.0 (red), H-chitosan-6.5 (orange), H-chitosan-7.0 (green), H-chitosan-7.5 (blue), H-chitosan-8.0 (light blue). Figure S14. Oscillating rotational rheometer measurements of chitosan (black), H-chitosan-6.0 (red), H-chitosan-6.5 (orange), H-chitosan-7.0 (green), H-chitosan-7.5 (blue), H-chitosan-8.0 (light blue). Figure S15. pH_B_ − pH of the blank value, which was measured by adding 30 mL of ultrapure water to the H-chitosan sample, pH_0_ is the initial pH of the CdSO_4_ solution, and pH_eq_ is the pH value of the solution after the adsorption process.
