# Supplementary material for: A Complementary and Revised View on the N-Acylation of Chitosan with Hexanoyl Chloride
Source: Mar Drugs. 2021 Jul 2;19(7):385. doi: 10.3390/md19070385 (PMC8303196; doi:10.3390/md19070385)
Supplement: Supplementary file 1 [file marinedrugs-19-00385-s001.zip › marinedrugs-1224890-supplementary.pdf]

marine drugs

Supporting Information

A Complementary and Revised View on the N-Acylation of Chitosan with Hexanoyl Chloride

Berthold Reis [1], Niklas Gerlach [1], Christine Steinbach [1], Karina Haro Carrasco[1], Marina Oelmann [1], Simona Schwarz [1], Martin Müller [1] and Dana Schwarz [1,*]

[1] Leibniz-Institut fuer Polymerforschung Dresden e.V., Hohe Straße 6, 01069 Dresden, Germany; reis@ipfdd.de (B.R.); gerlach@ipfdd.de (N.G.); steinbach@ipfdd.de (C.S.); haro@ipfdd.de (K.H.C.); oelmann@ipfdd.de (M.O.); simsch@ipfdd.de (S.S.); mamuller@ipfdd.de (M.M.)

[2] Department Chemistry and Food Chemistry, Technische Universität Dresden, 01062 Dresden, Germany

* Correspondence: schwarz-dana@ipfdd.de; Tel.: +49-351-46-58-542

Abstract: The modification of the biobased polymer chitosan is a broad and widely studied field. Herein an insight into the hydrophobization of low molecular weight chitosan by substitution of the amino functionalities with hexanoyl chloride is reported. Thereby the influence of the pH of the reaction media was investigated. Further, methods for the determination of the degree of substitution based on ^{1}H-NMR, FTIR, and potentiometric titration were compared and discussed regarding their accuracy and precision. ^{1}H-NMR was the most accurate method while FTIR and the potentiometric titration, though precise and reproducible, underlie the influence of complete protonation and solubility issues. Additionally, the impact of the pH variation during the synthesis on the properties of the samples was investigated by Cd^{2+} sorption experiments. The adjusted pH values during the synthesis and therefore the obtained degrees of substitution possessed a strong impact on the adsorption properties of the final material.

Keywords: hexanoyl chitosan; chitosan modification; substitution degree; pH variation

Table of Content

1. Experiments 3
1.1. Synthesis 3
2. Results 4
2.1. Side reactions 4
2.2. Characterization 4
2.1.1 ^{1}H-NMR spectra 4
2.1.2 ATR-FTIR spectra 6
2.1.3 Thermogravimetric analysis (TG) 10
2.1.4 Potentiometric titration of H-chitosans 11
2.1.5 DLS measurements 12
2.1.6 Nitrogen sorption measurements 15
2.1.7 X-ray diffraction measurements 16
2.1.7 Rheology measurements 17
2.2 Sorption Experiments 18
2.2.1 pH values 18

1. Experiments

1.1. Synthesis

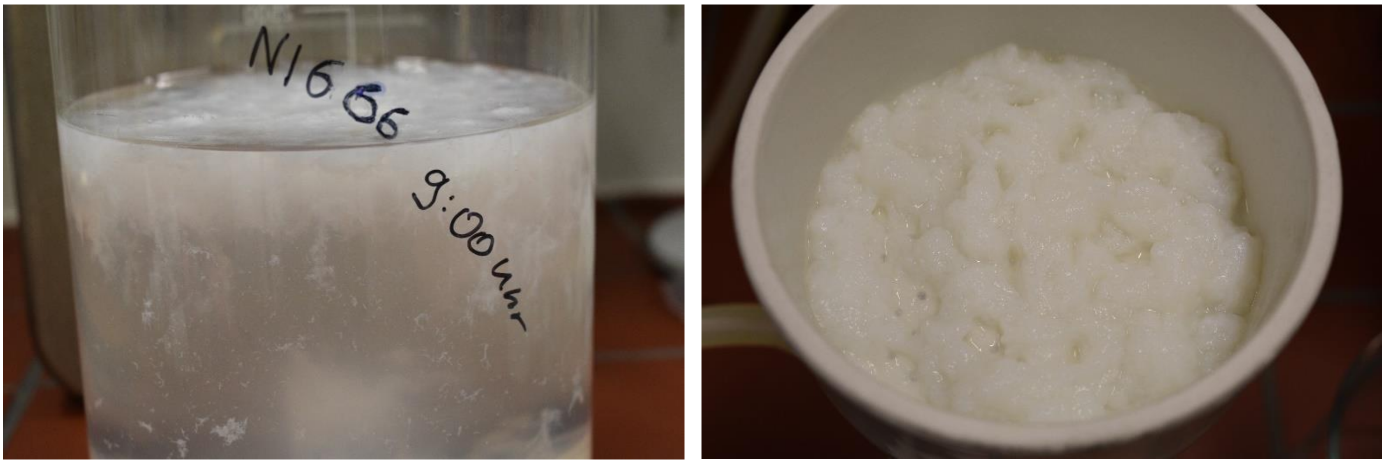

(**a**) (**b**)

Figure S1. Images of the precipitation in acetone (**a**) and the gel-like product after filtering and washing with methanol (60 °C) (**b**) (here H-chitosan-6.0).

(**a**) (**b**)

(**c**) (**d**)

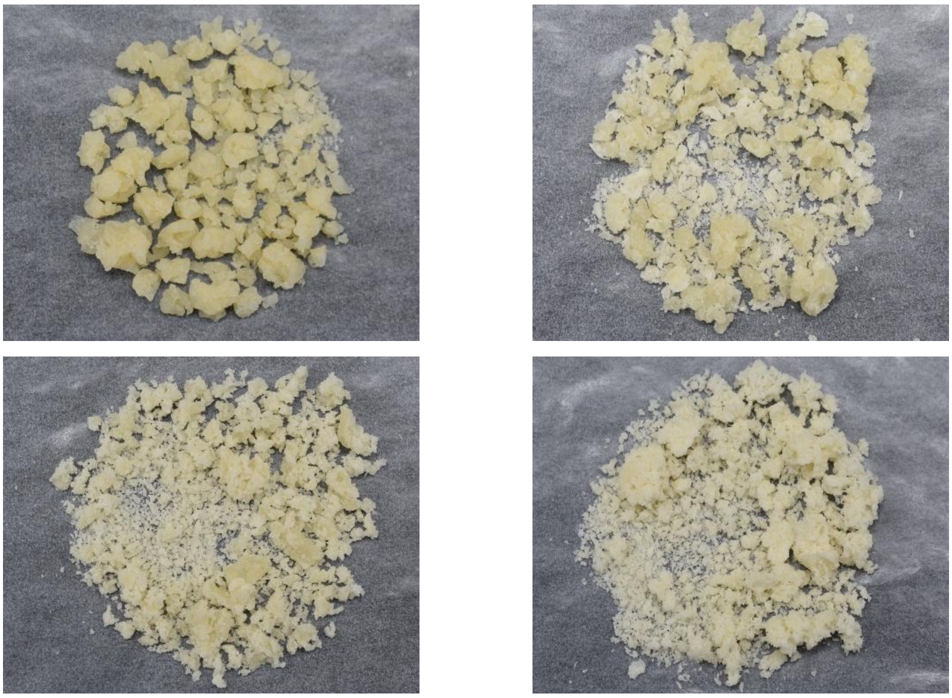

Figure S2. Images of the dried products H-chitosan-6.0 (**a**), H-chitosan-6.5 (**b**), H-chitosan-7.5 (**c**), H-chitosan-8.0 (**d**). The lower pH values tend to form more compact fragments.

2. Results

2.1. Side reactions

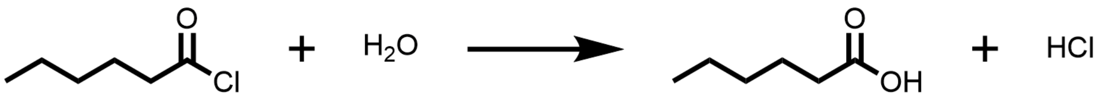

Scheme S1. Hydrolysis of hexanoyl chloride as competing reaction to chitosan acylation.

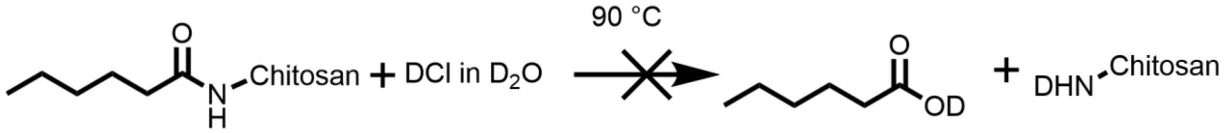

Scheme S2. Potential cleavage of hexanoyl functionality within the NMR tube (according to the NMR spectra, the reaction did not occur).

2.2. Characterization

2.1.1 ^{1}H-NMR spectra

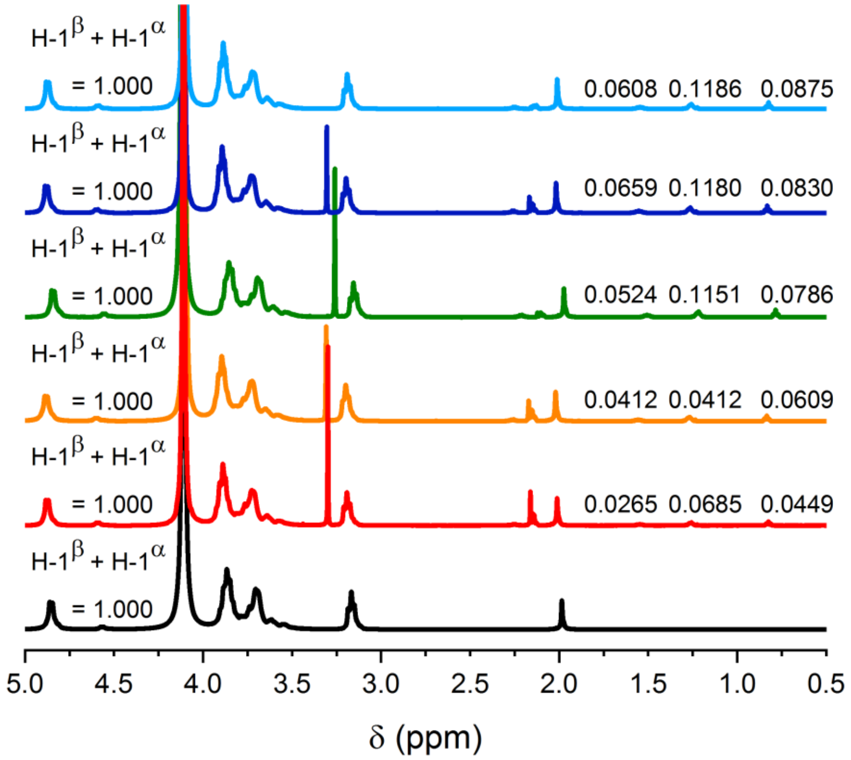

Figure S3. ^{1}H-NMR spectra of chitosan (black), H-chitosan-6.0 (red), H-chitosan-6.5 (orange), H-chitosan-7.0 (green), H-chitosan-7.5 (blue), H-chitosan-8.0 (light blue) and the respective integrals when H-1+ H′-1 = 1.000.

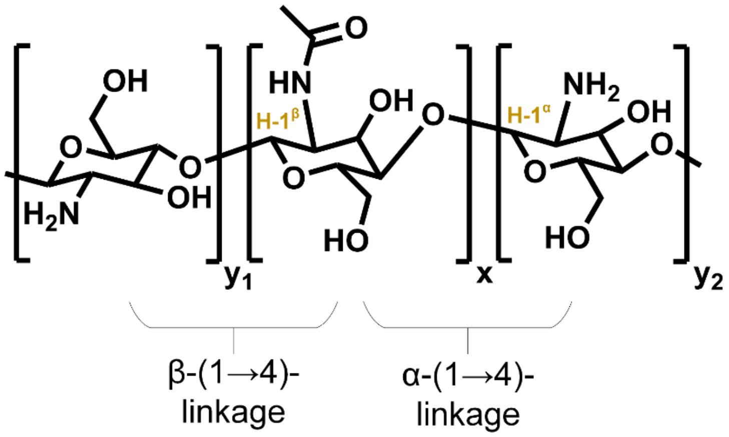

Scheme S3. Different glycosidic linkages within chitosan backbone leading to the two ^{1}H-NMR signals H-1$^{\beta}$ and H-1$^{\alpha}$.

2.1.2 ATR-FTIR spectra

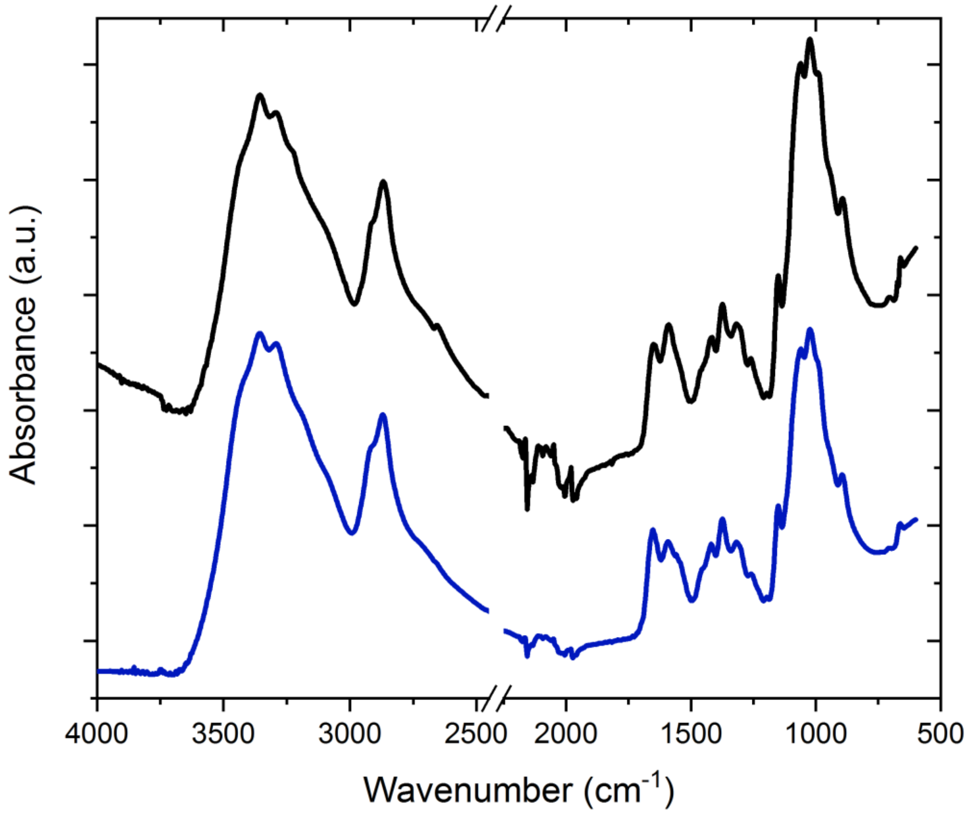

Figure S4. Powder ATR-FTIR spectra (method 1 , 100 scans, 2 cm^{-1} resolution) of native chitosan 90/60/A1 (black) and H-chitosan-7.5 (blue).

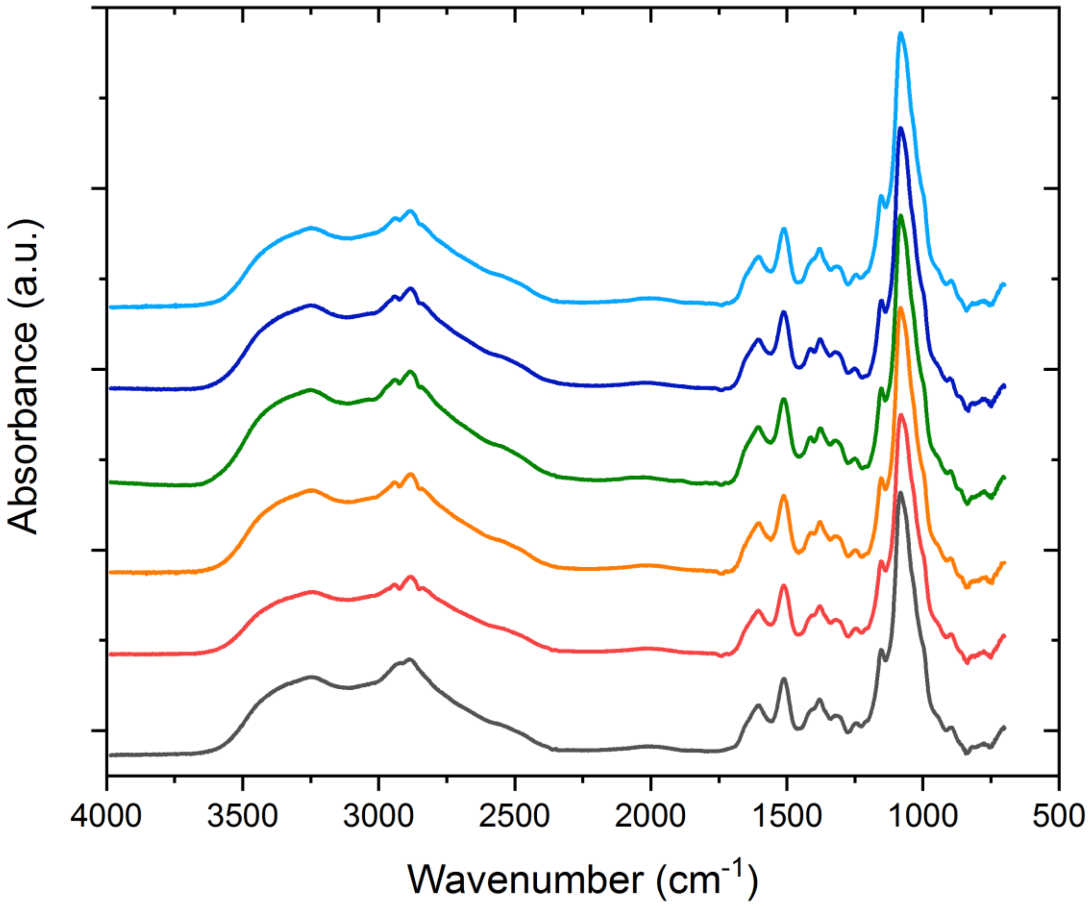

Figure S5. Thin film ATR-FTIR spectra (method 2, 100 scans, 2 cm^{-1} resolution) of chitosan (black), H-chitosan-6.0 (red), H-chitosan-6.5 (orange), H-chitosan-7.0 (green), H-chitosan-7.5 (blue), H-chitosan-8.0 (light blue).

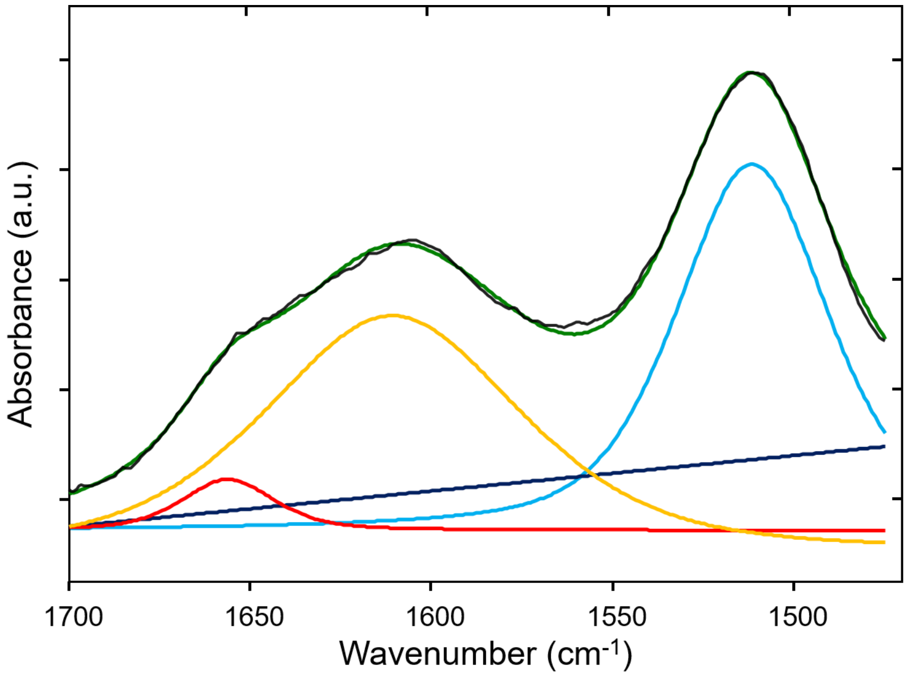

Figure S6. Line shape analysis of H-chitosan-7.5 with thin film measured spectrum (black), fitted spectrum (green, rms = 0.00088) using a sum of the three components at 1656 cm^{-1} (Amide I, red), 1611 cm^{-1} (yellow) and 1511 cm^{-1} (δNH_3^+, light blue) and baseline (dark blue).

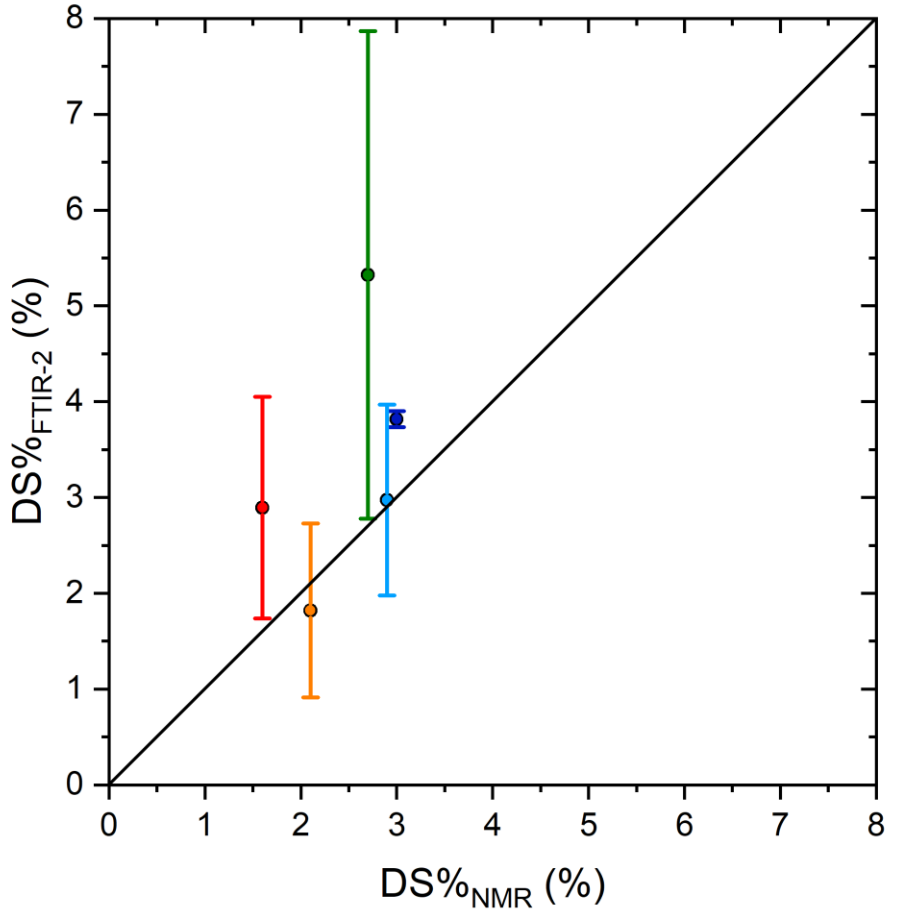

Figure S7. Correlation of DS%$_{FTIR-2}$ (error bars represent standard deviation of repeated measurements) and DS%$_{NMR}$ with a maximum absolute deviation of 2.6% and an average deviation of 1.0%. DS%$_{FTIR-2}$ tends to render higher DS% than those determined via NMR. The black line indicates ideal correlation of DS%$_{FTIR-2}$ and DS%$_{NMR}$. H-chitosan-6.0 (red), H-chitosan-6.5 (orange), H-chitosan-7.0 (green), H-chitosan-7.5 (blue), H-chitosan-8.0 (light blue).

2.1.3 Thermogravimetric analysis (TG)

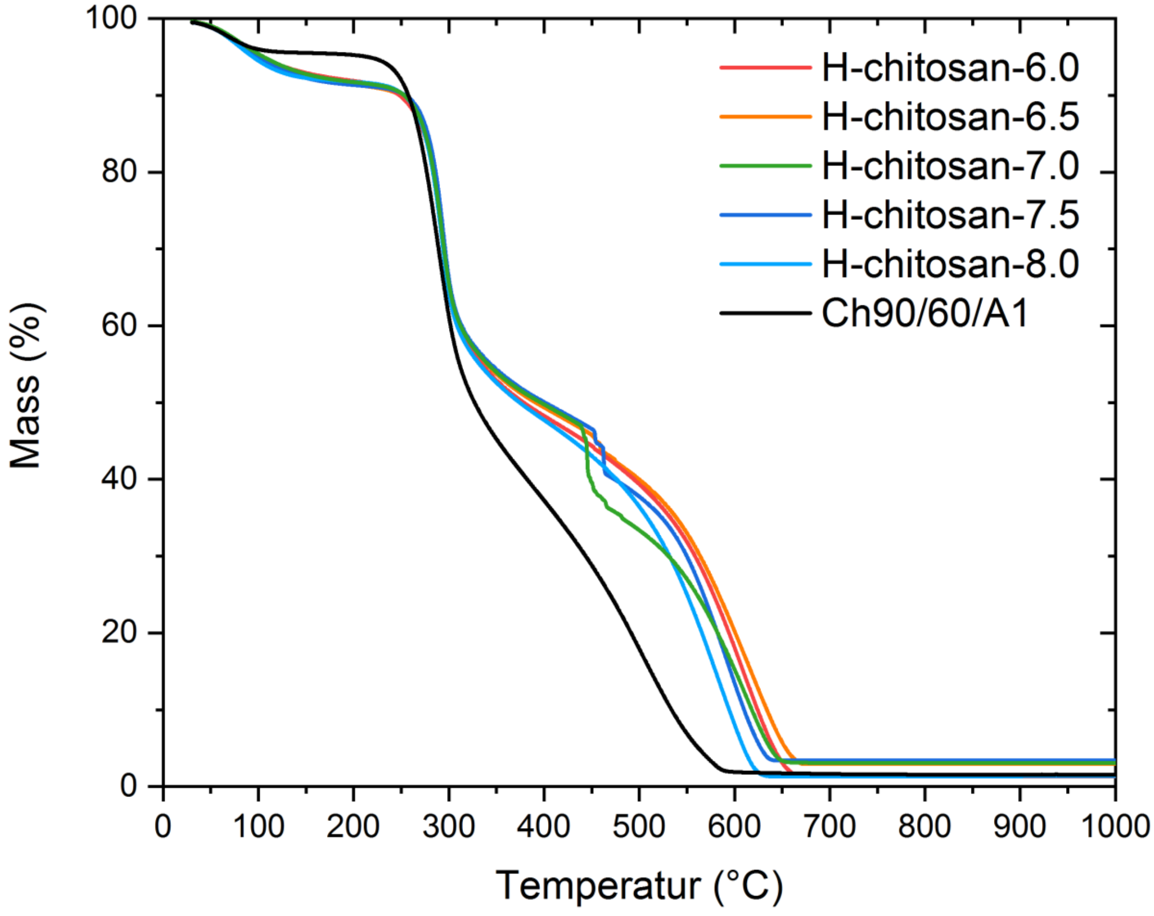

Figure S8. Thermogravimetric analysis of chitosan (black), H-chitosan-6.0 (red), H-chitosan-6.5 (orange), H-chitosan-7.0 (green), H-chitosan-7.5 (blue), H-chitosan-8.0 (light blue) under air atmosphere.

2.1.4 Potentiometric titration of H-chitosans

Table SI 1. Substitution degrees of H-chitosans determined via potentiometric titration (solution method 24 h at r.t.) and difference to $DS\%_{NMR}$.

H-chitosan	$DS\%_{Pot-1}$	$\Delta(DS\%_{Pot-1} - DS\%_{NMR})$
H-chitosan-6.0	8.2%	6.6%
H-chitosan-6.5	9.8%	7.7%
H-chitosan-7.0	10.8%	8.1%
H-chitosan-7.5	8.6%	5.6%
H-chitosan-8.0	8.5%	5.6%

2.1.5 DLS measurements

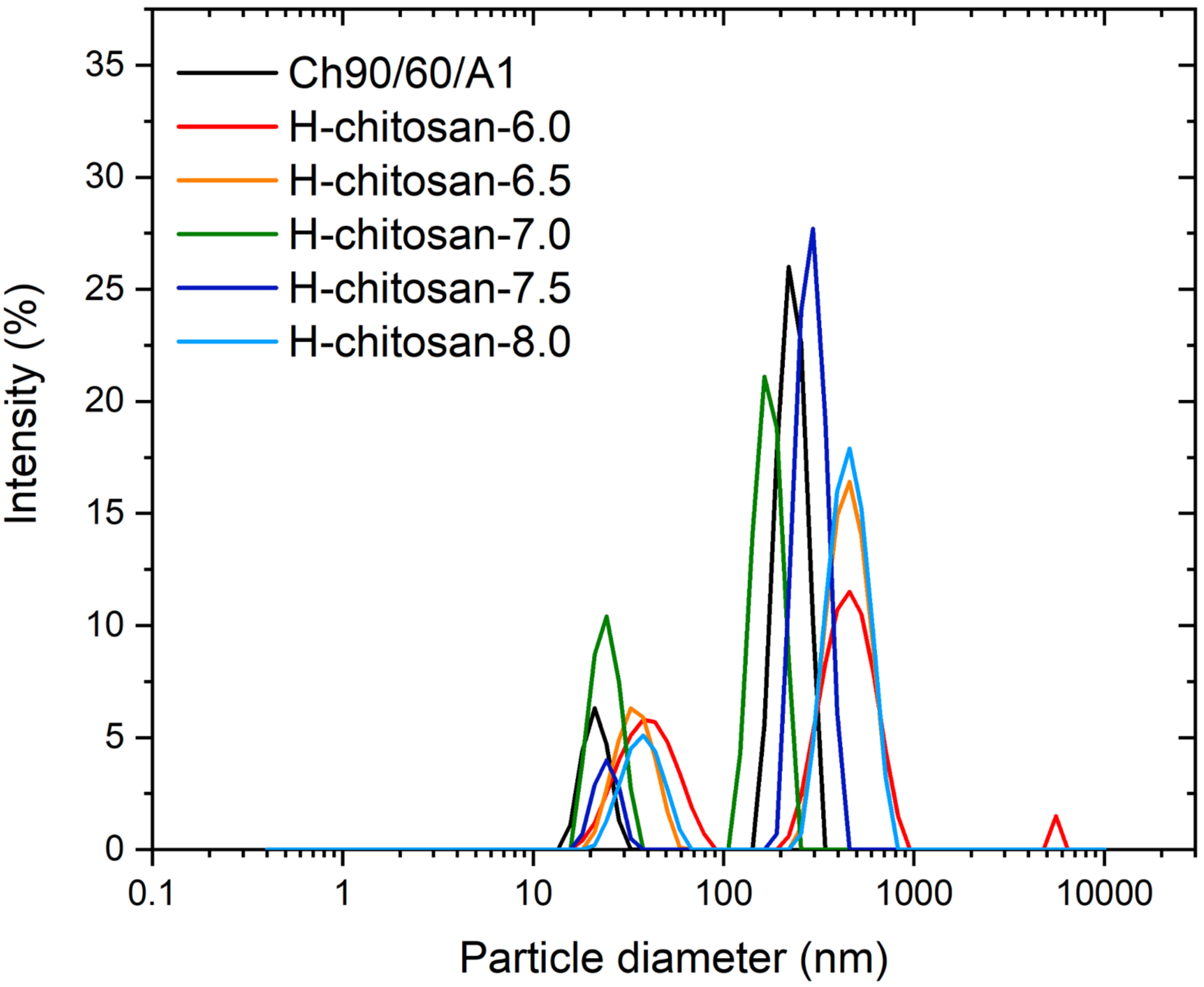

Figure S9. DLS intensity distribution measurements (1 g/L, pH 1, solved at r.t. for 24 h) of native chitosan (black), H-chitosan-6.0 (red), H-chitosan-6.5 (orange), H-chitosan-7.0 (green), H-chitosan-7.5 (blue), H-chitosan-8.0 (light blue). In all H-chitosan solutions, large particles visible by naked eye were in the solution but too large in order to be measured with DLS.

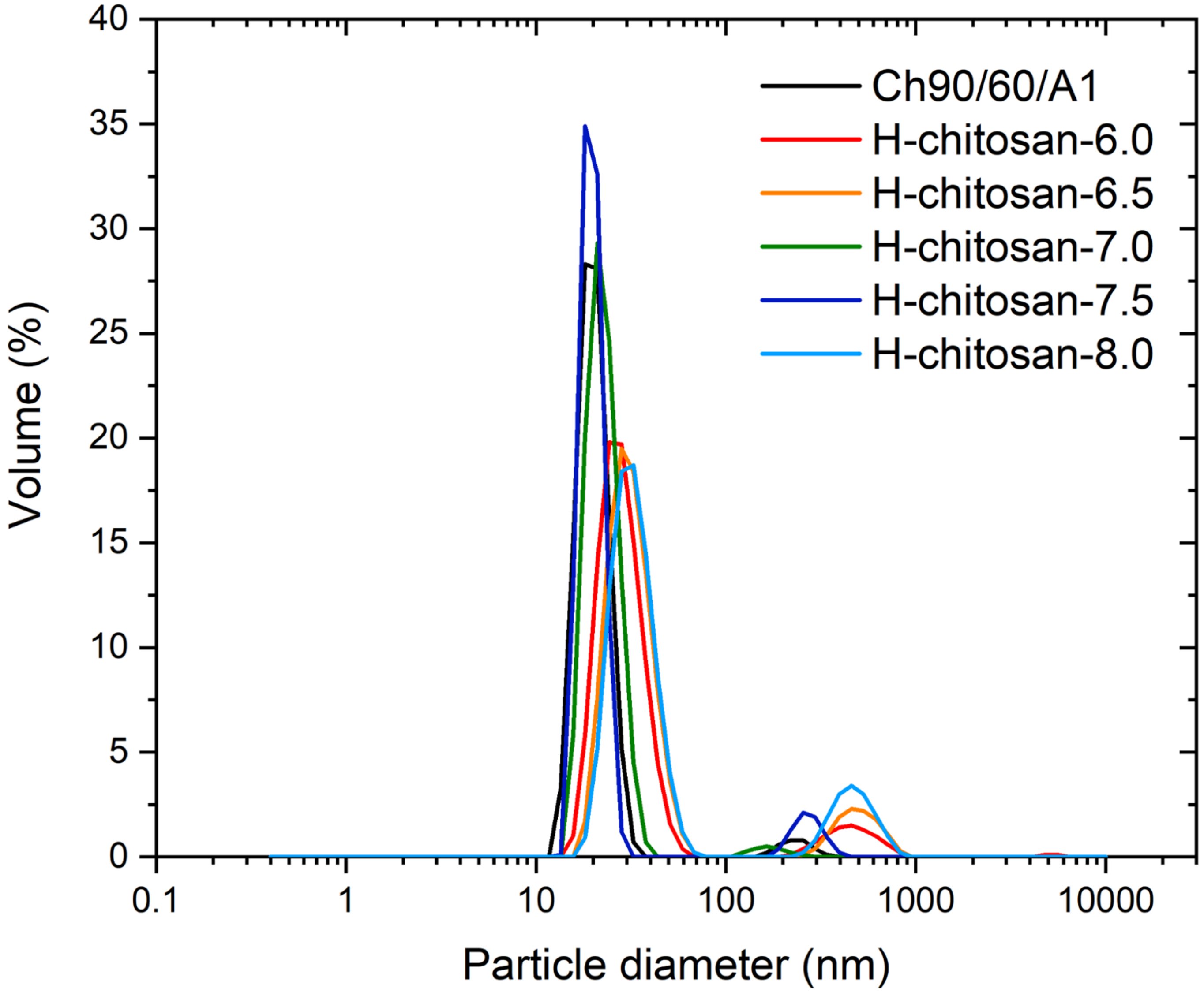

Figure S10. DLS volume distribution measurements (1 g/L, pH 1, solved at r.t. for 24 h) of native chitosan (black), H-chitosan-6.0 (red), H-chitosan-6.5 (orange), H-chitosan-7.0 (green), H-chitosan-7.5 (blue), H-chitosan-8.0 (light blue). In all H-chitosan solutions, small, unsolved particles were visible, that are too large in order to be measured with DLS.

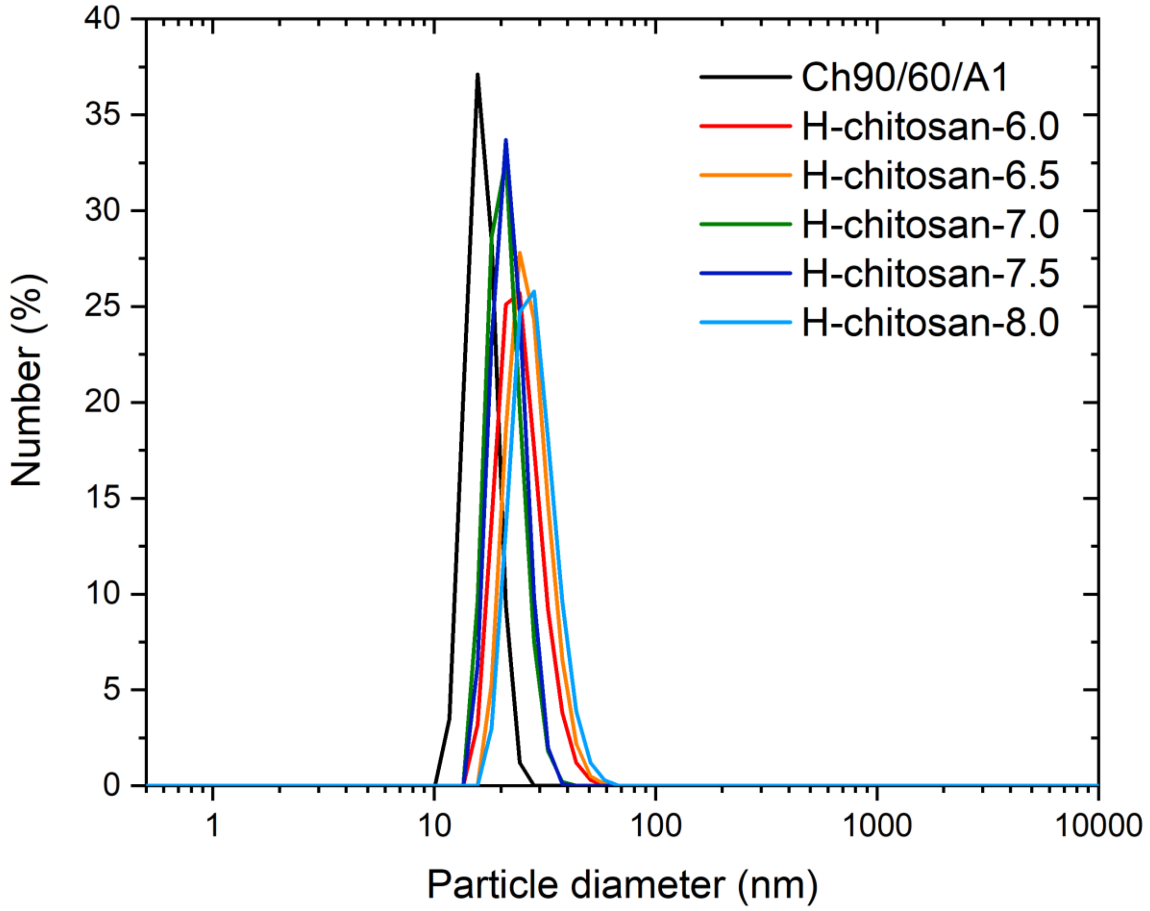

Figure S11. DLS number distribution measurements (1 g/L, pH 1, solved at r.t. for 24 h) of native chitosan (black), H-chitosan-6.0 (red), H-chitosan-6.5 (orange), H-chitosan-7.0 (green), H-chitosan-7.5 (blue), H-chitosan-8.0 (light blue). In all H-chitosan solutions, small, unsolved particles were visible, that are too large in order to be measured with DLS.

2.1.6 Nitrogen sorption measurements

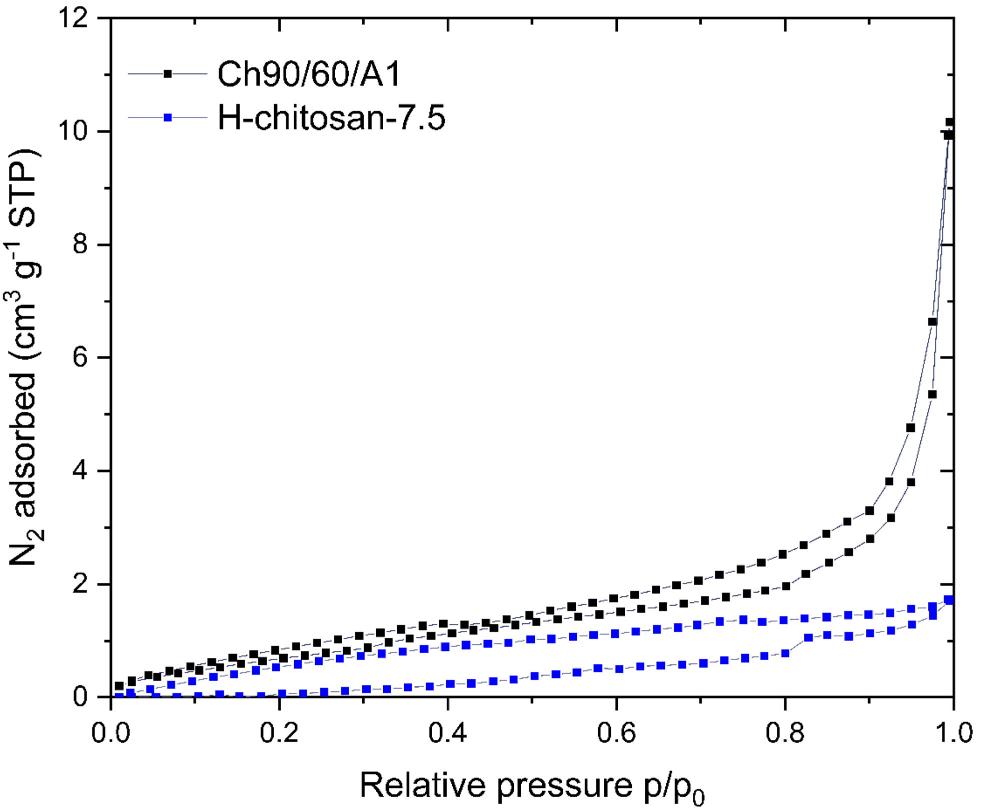

Figure S12. Nitrogen sorption measurements. Neither native chitosan 90/60/A1 (black) nor H-chitosan (e.g. H-chitosan-7.5, blue) exhibited significant specific surface areas ($S_{BET} < 3$ m²/g).

2.1.7 X-ray diffraction measurements

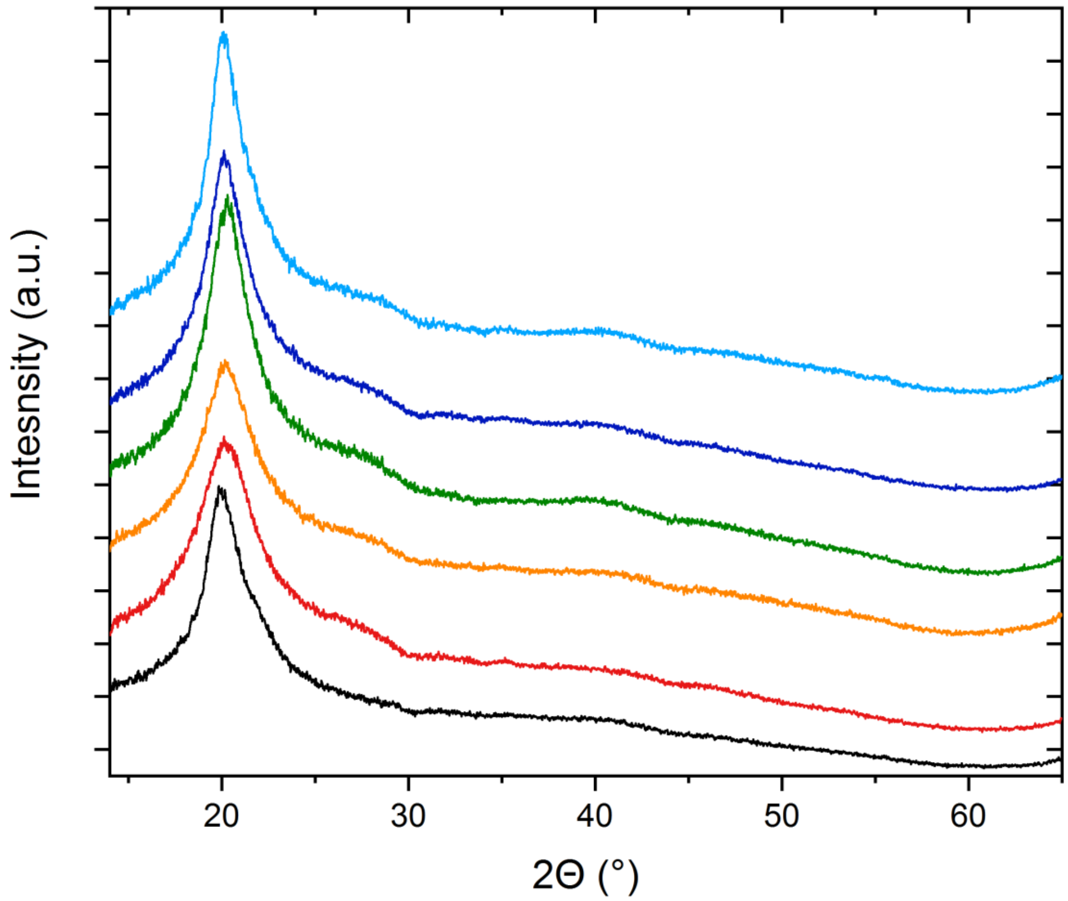

Figure S13. X-ray diffraction measurements of chitosan (black), H-chitosan-6.0 (red), H-chitosan-6.5 (orange), H-chitosan-7.0 (green), H-chitosan-7.5 (blue), H-chitosan-8.0 (light blue).

2.1.7 Rheology measurements

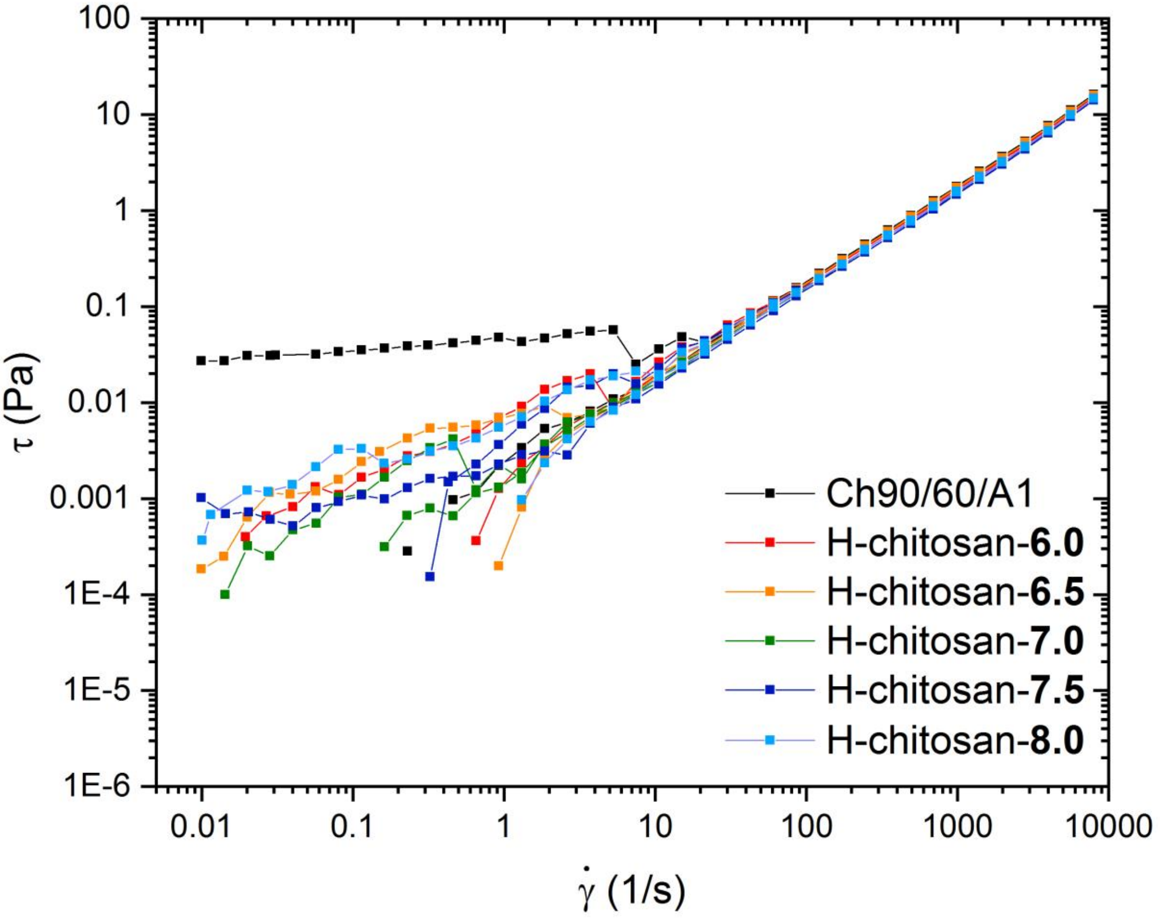

Figure S14. Oscillating rotational rheometer measurements of chitosan (black), H-chitosan-6.0 (red), H-chitosan-6.5 (orange), H-chitosan-7.0 (green), H-chitosan-7.5 (blue), H-chitosan-8.0 (light blue).

2.2 Sorption Experiments

2.2.1 pH values

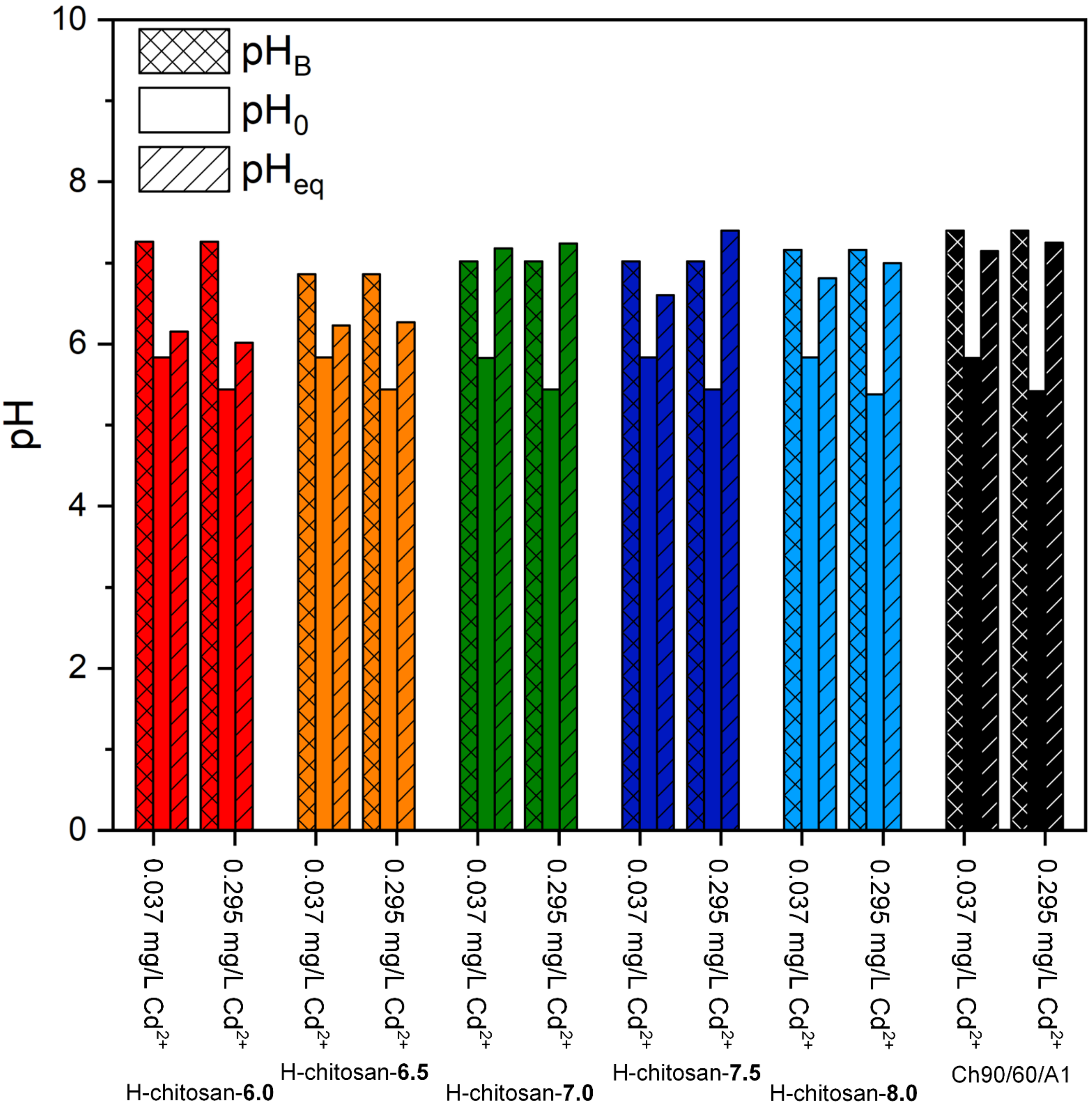

Figure S15. pH_B – pH of the blank value, which was measured by adding 30 mL of ultrapure water to the H-chitosan sample, pH_0 is the initial pH of the $CdSO_4$ solution and pH_{eq} is the pH value of the solution after the adsorption process.
